# E-Cigarettes are More Addictive than Traditional Cigarettes—A Study in Highly Educated Young People

**DOI:** 10.3390/ijerph16132279

**Published:** 2019-06-27

**Authors:** Mateusz Jankowski, Marek Krzystanek, Jan Eugeniusz Zejda, Paulina Majek, Jakub Lubanski, Joshua Allan Lawson, Grzegorz Brozek

**Affiliations:** 1 Department of Epidemiology, School of Medicine in Katowice, Medical University of Silesia in Katowice, Medykow 18 Str, 40-752 Katowice, Poland; 2 Department of Psychiatric Rehabilitation, Department of Psychiatry and Psychotherapy, School of Medicine in Katowice, Medical University of Silesia in Katowice, Ziolowa 45/47 Str, 40-635 Katowice, Poland; 3 Canadian Centre for Health and Safety in Agriculture, College of Medicine, University of Saskatchewan, 104 Clinic Place, P.O. Box 23, Saskatoon, SK S7N 2Z4, Canada; 4 Department of Medicine, College of Medicine, University of Saskatchewan, 103 Hospital Drive, Saskatoon, SK S7N 0W8, Canada

**Keywords:** electronic cigarettes, tobacco, nicotine, addiction, dependence

## Abstract

E-cigarettes are often considered less addictive than traditional cigarettes. This study aimed to assess patterns of e-cigarette use and to compare nicotine dependence among cigarette and e-cigarette users in a group of highly educated young adults. From 3002 healthy adults, a representative group of 30 cigarette smokers, 30 exclusive e-cigarette users, and 30 dual users were recruited. A 25-item questionnaire was used to collect information related to the patterns and attitudes towards the use of cigarettes and e-cigarettes. The Fagerström test for nicotine dependence (FTND) and its adapted version for e-cigarettes were used to analyze nicotine dependence in each of the groups. The nicotine dependence levels measured with FTND were over two times higher among e-cigarette users (mean 3.5) compared to traditional tobacco smokers (mean 1.6; *p* < 0.001). Similarly, among dual users, nicotine dependence levels were higher when using an e-cigarette (mean 4.7) compared to using traditional cigarettes (mean 3.2; *p* = 0.03). Habits and behaviors associated with the use of e-cigarettes did not differ significantly (*p* > 0.05) between exclusive e-cigarette users and dual users. The findings suggest that e-cigarettes may have a higher addictive potential than smoked cigarettes among young adults.

## 1. Introduction

Nicotine is a highly addictive substance and adolescents and young adults may be at high risk of addiction [1,2]. Nicotine withdrawal effects such as irritability, anxiety, poor concentration, memory impairment, and insomnia can occur after cessation of chronic nicotine use [3]. In addition, exposure to nicotine in adolescence may have adverse affects on the adolescent brain, with postulated risks including impaired memory and attention, increased risk of substance abuse, and addiction and poor academic performance [4,5,6].

Electronic cigarettes (e-cigarettes) are a relatively new form of nicotine delivery device and the prevalence of their use is rapidly growing in many countries [7,8,9]. E-cigarettes have been suggested as a way to reduce nicotine dependence and have been reported to be less addictive than traditional cigarettes [10]. However, there have been numerous reports of people who become addicted to e-cigarettes and report typical symptoms of nicotine addiction [10,11,12], including with a dose-response effect [13]. E-cigarette use has even been suggested to lead to an increased risk of addiction, especially among young people [14], with higher exposure to doses of nicotine compared to people who smoke traditional cigarettes, as e-cigarettes are used in times and places where smoking is prohibited [15]. While some suggest e-cigarettes will help to quit smoking or reduce this addiction [16], others have reported that using e-cigarettes has a negative impact on successfully quitting [17,18,19]. Using e-cigarettes may have little effect on reducing the frequency of nicotine use [20,21].

Due to uncertainty about the dependency potential of e-cigarettes, we decided to carry out a study aimed to: (1) assess the patterns of e-cigarette use by exclusive e-cigarette users and dual users and (2) compare nicotine dependence levels among young adults who use cigarettes or e-cigarettes. We chose to examine the problem in a highly educated group of university students under the assumption that their perception is less biased by commonly held beliefs and stereotypes of e-smoking safety and addiction mechanisms.

## 2. Materials and Methods

### 2.1. Participants

This study is a continuation of a survey-based and multicentered international project, the YoUng People E-Smoking Study (YUPESS) [22]. From this larger study, we selected a subgroup to be involved in the current study.

A cross-sectional survey was performed between January and March 2018. Three fields of study (medical, humanistic, and technical) were randomly selected from a group of 245 different fields of study within Universities in Katowice. The questionnaire was addressed to all the students within each selected field of study. Data were collected from 3002 participants. Out of all the participants, we identified 39 exclusive e-cigarette users and 54 dual users. All subjects who used e-cigarettes (*n* = 93) were invited to participate in the second part of the study. Exclusion criteria included occurrence of any chronic diseases, history of lung diseases (e.g., asthma or bronchial hyperactivity in childhood), presence of any allergic diseases, any medication intake (current or in the last 2 weeks), acute illnesses or infections in the last 2 weeks, influenza vaccination in the last 2 weeks, and current pregnancy or lactation. Among exclusive e-cigarette users, three met the exclusion criteria and six refused. Finally, 30 exclusive e-cigarette users were included. Among dual users, four met the exclusion criteria. To keep an equal number of participants per group, out of 50 eligible dual users, we randomly selected a group of 30 dual users, using randomization procedures available in the Statistica package. An additional group of 30 people was drawn randomly from a group of cigarette smokers (*n* = 433).

### 2.2. Measures

During the preparation of the study protocol, we analyzed the currently available data on nicotine addiction among e-cigarette users [23,24,25]. The questionnaire from the cross-sectional study was used to assess the behavioral aspects of cigarette and e-cigarette smoking and included 25 items (items and content are located in Supplementary Materials File S1) related to traditional and electronic cigarettes. The Fagerström test for nicotine dependence (FTND) [26] and its adapted versions for e-cigarettes [23,27] were used to measure nicotine dependence. The scores from each of the six questions of the FTND were summed and an overall total score for nicotine dependence was calculated. All the questionnaires were administered in person to each of the participants.

Validity and reliability of the questionnaire was tested in a pilot study of 28 students. First, content validity was considered acceptable based on a literature review [23,24,25] and scientific discussion during a research team meeting and conference presentation. Second, reliability was assessed by having the students complete the survey, twice, five days apart. Cronbach’s alpha for the survey items ranged between 0.76 to 0.98. Participation in the study was voluntary and anonymous. The study protocol was reviewed and approved by the Ethical Review Board at the Medical University of Silesia, Poland (consent number: KNW/0022/KB1/37/I/17).

### 2.3. Statistical Analysis

Data were analyzed using Statistica 12 Software (TIBCO Software Inc., Palo Alto, CA, USA). Statistical significance of differences between continuous variables was analyzed using an independent samples t-test or a Mann-Whitney U test where appropriate. Statistical testing to compare categorical variables was completed using an independent samples chi-square test. We assessed strength of association by calculating odds ratios (OR). We used 95% confidence intervals (CI) and defined statistical significance as *p* < 0.05.

## 3. Results

Of the adults included (39.8% female; mean age 22.4 ± 2.2 years old), the mean smoking duration was 50.0 ± 32.0 months among smokers and 67.3 ± 30.5 months among dual users (*p* = 0.03). Duration of e-cigarette use was comparable between exclusive e-cigarette users and dual users being 29.0 ± 24.1 and 27.7 ± 17.4 months, respectively (*p* = 0.6). There were no statistically significant differences in sex, age, or the daily number of cigarettes or e-cigarette smoking sessions between groups (*p* > 0.05).

### 3.1. Behavioral Aspects and Patterns of E-Cigarette Use

Habits and behaviors associated with the use of e-cigarettes, including nicotine content in the e-liquid, e-liquid consumption, type of e-liquid used, and the number of e-cigarettes used, did not differ significantly (*p* > 0.05) between e-cigarette users and dual users. Users of e-cigarettes (*n* = 60) consumed an average of 4.2 mL of e-liquid per day, with the most frequently chosen e-liquid being that containing 6 mg of nicotine in 1 mL of e-liquid. None of the subjects used nicotine free e-liquids. The study population was dominated by individuals who prepared e-liquids themselves: 66.7% of e-cigarette users and 74.1% of dual users (Table 1). Over half (52.7%) of the e-cigarette users (53.6% of exclusive e-cigarette users and 51.9% dual users; *p* = 0.9) had chosen devices that allow technical modifications of the e-cigarette such as voltage, power, and resistance of the heater.

**Figure 1:**
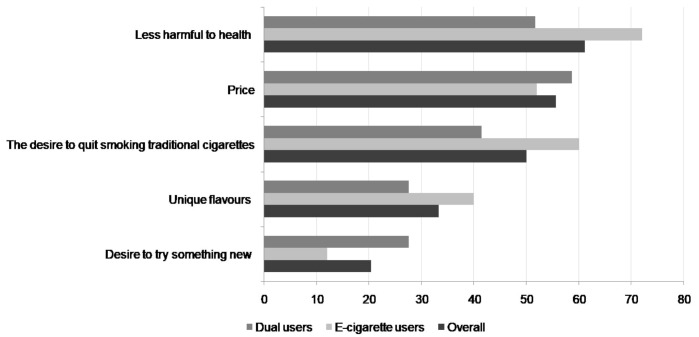
Motivations for e-cigarette use.

Almost all exclusive e-cigarette users (96%) had ever tried cigarette smoking. Out of the 60 e-cigarette users, 18.5% initiated nicotine use by e-cigarette. E-cigarette, as a first nicotine product, was declared by 16% of exclusive e-cigarette users and 20.7% of dual users (*p* = 0.6). The most common reasons stated for starting to use e-cigarettes were it being less harmful to health, price, and desire to quit traditional smoking. The reasons stated for using e-cigarettes are presented in Figure 1.

Dual users who were actively smoking both types of cigarettes (traditional and e-cigarette) indicated that traditional cigarette smoking offers more smoking satisfaction (46.2%) compared to using e-cigarettes (23%). Also, smoking a cigarette in a group of friends, in combination with a cup of coffee, after a meal, or after sex was associated with a greater subjective feeling of satisfaction compared to the feelings associated with the use of an e-cigarette in the same situations. Dual users indicated that the smell (92.9%) and the taste (82.1%) of e-cigarettes as well as the subjective feeling associated with the inhalation and exhalation of vapor generated from e-cigarettes were the primary advantages of e-cigarettes over traditional cigarettes.

### 3.2. Fagerstrom Scores and Levels of Nicotine Addiction

The average FTND score among exclusive e-cigarette users was over twice as high (mean 3.5 versus 1.6) as among traditional cigarette smokers (*p* = 0.002; Table 2). The mean nicotine dependence level from e-cigarettes (mean 4.7) was higher than that from traditional cigarettes (mean 3.2; *p* = 0.03) among dual users. E-cigarette users were more likely to use an e-cigarette in the first 30 min after waking and were more likely to find it difficult to refrain from using e-cigarettes in places where it is forbidden (Table 2).

## 4. Discussion

The results of our study allow us to characterize patterns of use of e-cigarettes among young adults in Poland who are experienced users of e-cigarettes. Almost one-fifth of the participants initiated nicotine use bye-cigarette. Most subjects started e-cigarette use due to health-related (harm reduction or for smoking cessation) or financial reasons. The participants consumed relatively large amounts of nicotine-containing e-liquid (almost half a bottle per day). More than half of the participants chose technically advanced e-cigarette devices.

Our key finding was that nicotine dependence levels measured with FTND were over two times higher among exclusive e-cigarette users (mean 3.5) compared to traditional tobacco smokers (mean 1.6; *p* < 0.001). Similarly, among dual users, nicotine dependence levels were higher when using an e-cigarette (mean 4.7) compared to a traditional cigarette (mean 3.2; *p* = 0.03). The high levels of nicotine dependence observed among young adult e-cigarette users compared to smokers in our study suggest this group may be at high risk of addiction.

We also found that dual users used e-liquids with higher concentrations of nicotine and consumed more milliliters of e-liquid per day compared to exclusive e-cigarette users. These findings together with the higher mean FTND score suggests a higher nicotine dependence level in this group of e-cigarette users. However, by contrast exclusive e-cigarette users presented with a longer duration of e-cigarette use (on average 1.3 months longer) compared to dual users, although this was not statistically significant.

Our findings contrast with results obtained by González et al. [28], Etter et al. [23], and Liu et al. [29]. In a study conducted by González et al., e-cigarette users scored lower in FTND (mean 4.38) than cigarette smokers (mean 5.57; *p* = 0.03) [28]. Etter et al. concluded that e-cigarettes are less addictive than tobacco cigarettes and their addictive potential is comparable to nicotine gums [23], although they also found that users of nicotine-containing e-cigarettes presented higher dependence ratings compared to the users of nicotine-free e-cigarettes [23]. When comparing nicotine dependence among exclusive e-cigarette and cigarette users, Liu et al. showed that among smokers, time to the first cigarette after waking is shorter (20 min) compared to people using e-cigarettes (29.2 min) [29]. Also, people who smoke cigarettes more often consider themselves addicted, with more reporting a strong compulsion to smoke. On this basis, the researchers stated that e-cigarettes are less addictive [29].

Several hypotheses should be considered to explain why we found higher levels of addiction among e-cigarettes users compared to smokers in contrast to findings in studies by González et al. (2017) [28], Etter et al. (2015) [23], and Liu et al. (2017) [29]. First, our study was performed in early 2018, and it is possible that our participants were using more effective e-cigarettes with higher addictive potential. The e-cigarette market is quickly changing with newer models and generations of e-cigarettes available. Advanced e-cigarettes, especially those with a high-capacity battery or accumulator, are characterized by the production of a larger volume of aerosol and delivery of significantly higher doses of nicotine compared to older models (first or second generation) of e-cigarettes [30]. In our study, participants, on average, used four different e-cigarette devices, and more than half of the participants used technically advanced e-cigarettes. Second, our study was performed among young adult e-cigarette users, aged 22.4 ± 2.2 years old, a younger study population compared to those of earlier studies performed by González et al. [28], Etter et al. [23], and Liu et al. [29]. The patterns of e-cigarette use by young adults differ from those of older populations of e-cigarettes users [24,31]. It should also be noted that patterns of smoking may differ and older smokers tend to smoke more cigarettes per day compared to adolescents, and may therefore have higher FTND scores. However, adolescents and young adults (under 25 years) are also at the greatest risk of nicotine addiction [2]. These factors could have contributed to higher FTND scores being found among e-cigarette users than among smokers in our study compared to previously published studies [23,28,29]. Third, studies by González et al. (2017) [28], Etter et al. (2015) [23], and Liu et al. (2017) [29], had different designs and larger sample sizes compared to our study, which could also have affected the results.

In our study, e-cigarette users consumed an average of 4.2 mL of e-liquid per day. Slightly lower results were obtained by Hajek et al. at 3.3 mL per day [31] and Dawkins et al. at 3.4 mL [24] of e-liquid per day. In the current study, 70% of all e-cigarette users had used self-prepared e-liquids. This percentage is more than twice as high as that described in the literature (30.8% by Wong et al. [32] and 32.9% by Farsalinos et al. [33]). Such a high percentage of people preparing e-liquids themselves may contribute to the high consumption of e-liquids as self-preparation of e-liquids is a way to reduce costs of e-cigarette use, especially in such a young population of e-cigarette users.

Strengths of this study include the detailed description of patterns of use of young, experienced e-cigarette users and the investigation of the degree of addiction of e-cigarettes and cigarettes using an established measure of addiction among a key population group—young adults. Further separation of this group into groups of exclusive e-cigarette users and dual users allowed for a fuller understanding of the differences in behaviors and addiction presences between these groups.

There are several potential limitations of the current study. First, our study was limited to a small sample of young e-cigarette users from one region of Poland. This is a specific group of e-cigarette users which was identified among the 3002 young adults who participated in an across-sectional survey (YUPESS—Katowice branch), with the sample being representative of young adults with higher education (university students) in Poland [34] but not of non-university-attending young adults. Secondly, nicotine dependence levels were not confirmed biochemically. Measurement of nicotine in blood or cotinine in urine or saliva could provide more detailed information on nicotine dependence and exposure to cigarette smoke. However, self-declared smoking statuses were confirmed by measured levels of exhaled carbon monoxide (CO), which has been described in another article [35]. The daily number of cigarettes smoked by the smokers group was lower compared to the general population. However, we are aware that our study is limited to a group of young, educated adults and the smoking habits in such a group may differ compared to the general population, meaning our results should not be generalized to the whole population of e-cigarette users. Moreover, follow-up studies are needed to examine whether the high Fagerström scores obtained in our study translate into a high risk of long-term e-cigarette use. This will also allow us to better understand the natural history of smoking and e-cigarette use as well as dual use.

E-cigarettes are promoted as a safer alternative to traditional cigarettes. This view is also shared by e-cigarette users [8,36]. Exclusive e-cigarette users perceive e-cigarettes to be less harmful than people using traditional cigarettes or dual users [36]. Studies performed in New Zealand and Germany have shown that there is a public belief that e-cigarettes can help in quitting smoking [37,38]. Our findings suggest that the use of e-cigarettes carries a great risk of addiction and the risk of addiction may be even higher than that from smoked tobacco cigarettes, at least among young adults. The higher Fagerström scores among dual users could suggest that these individuals are at particularly high risk of addiction. In jurisdictions adopting a harm-reduction approach to tobacco control, balanced regulatory approaches may be needed that seek to minimise the availability and use of e-cigarettes among adolescents and young adults, whilst ensuring access to established smokers who wish to use e-cigarettes to help quit or as substitutes for cigarettes.

## 5. Conclusions

In conclusion, in this work the use of e-cigarettes among young adults was shown to result in higher nicotine dependence levels than nicotine dependence related to tobacco cigarette use. In addition, a stronger dependence on the e-cigarette compared to the traditional cigarette identified in dual users indicates that e-cigarettes may be highly addictive. Further research is necessary to provide evidence on the role of e-cigarettes in smoking cessation, especially in young adults. Social normalization of smoking behaviors by allowing e-cigarette use should be monitored.

## Figures and Tables

**Table 1:** Patterns and habits of e-cigarette use among e-cigarette users and dual users.

	Overall *n* = 60	Exclusive E-Cigarette Users *n* = 30	Dual Users *n* = 30	*p* *
**Average concentration of nicotine in the e-liquid [mg/mL]**				
Mean ± SD	7.5 ± 3.8	6.8 ± 3.4	8.3 ± 4.0	0.1
Median (min–max)	6.0 (2–18)	6.0 (2–12)	6.0 (3–18)	
**Milliliters of e-liquid used per day [mL]**				
Mean ± SD	4.2 ± 3.4	4.1 ± 3.9	4.3 ± 3.1	0.8
Median (min–max)	3.0 (0.15–15)	3.0 (0.3–15)	3.5 (0.15–10)	
**Milliliters of e-liquid used per month [mL]**				
Mean ± SD	107 ± 102.2	113.9 ± 121.2	100.5 ± 81.9	0.6
Median (min–max)	90.0 (10–550)	90.0 (10–550)	70.0 (10–300)	
**Type of e-liquid used**				
Purchased in the store % (95%CI)	29.6 (19.1–42.8)	33.3 (18.6–52.2)	25.9 (13.2–44.7)	0.6
Self-prepared % (95%CI)	70.4 (57.2–80.9)	66.7 (47.8–81.4)	74.1 (55.3–86.8)	
**Number of different models of e-cigarette (device) used**				
Mean ± SD	4 ± 4	3.6 ± 3.3	4.3 ± 4.6	0.5
Median (min–max)	3 (1–23)	3 (1–17)	3 (1–23)	

Legend: 95%CI, 95-percent confidence interval; min–max, minimum and maximum value range; * results of U Mann-Whitney test.

**Table 2:** Aspects of cigarette and e-cigarette smoking based on Fagerström test for nicotine dependence (FTND).

	Smokers *n* = 30 % (95%CI)	Exclusive E-Cigarette Users *n* = 30 % (95%CI)	Dual Users *n* = 30 % (95%CI)	*p*
**How soon after waking up do you reach for a cigarette?**				
Within 30 min	17.9 (7.9–35.6)		42.3 (25.5–61.1)	0.04
After 30 min	82.1 (64.4–92.1)		57.7 (38.9–74.5)	
**How soon after waking up do you reach for an e-cigarette?**				
Within 30 min		53.9 (35.5–71.2)	57.1 (39.1–73.5)	0.8
After 30 min		46.1 (28.8–64.5)	42.9 (26.5–60.9)	
**Do you find it difficult to refrain from smoking in places where it is forbidden?**				
Yes	10.7 (3.7–27.2)		19.2 (8.5–37.9)	0.4
No	89.3 (72.8–96.3)		80.8 (62.1–91.5)	
**Do you find it difficult to refrain from use e-cigarette in places where it is forbidden?**				
Yes		34.6 (19.4–53.8)	42.9 (26.5–60.9)	0.5
No		65.4 (46.2–80.6)	57.1 (39.1–73.5)	
**Which cigarette would you hate most to give up?**				
The first one in the morning	57.1 (39.1–73.5)		73.1 (53.9–86.3)	0.2
Any other	42.9 (26.5–60.9)		26.9 (13.7–46.1)	
**Which e-cigarette would you hate most to give up?**				
The first one in the morning		30.8 (16.5–50.0)	35.7 (20.7–54.2)	0.7
Any other		69.2 (50.0–83.5)	64.3 (45.8–79.3)	
**How many cigarettes per day do you smoke?**				
10 or less	85.7 (68.5–94.3)		69.2 (50.0–83.5)	0.2
11 to 20	14.3 (5.7–31.5)		23.1 (11.0–42.1)	
21–30	0.0 (0.0–11.3)		7.7 (2.1–24.1)	
31 or more	0.0 (0.0–11.3)		0.0 (0.0–11.3)	
**How many times a day do you use e-cigarettes? (Number of e-smoking sessions: one e-smoking session consist of 15 puffs or approximately 10 min of use)**				
10 or less		38.5 (22.4–57.5)	32.1 (17.9–50.7)	0.8
11 to 20		38.5 (22.4–57.5)	35.7 (20.7–54.2)	
21–30		11.5 (4.0–28.9)	10.7 (3.7–27.2)	
31 or more		11.5 (4.0–28.9)	21.4 (10.2–39.5)	
**Do you smoke more frequently during the first hours after waking than during the rest of the day?**				
Yes	14.3 (5.7–31.5)		34.6 (19.4–53.8)	0.08
No	85.7 (68.5–94.3)		65.4 (46.2–80.6)	
**Do you use e-cigarettes more frequently during the first hours after waking than during the rest of the day?**				
Yes		15.4 (6.2–33.5)	39.3 (23.6–57.6)	0.05
No		84.6 (28.8–64.5)	60.7 (42.4–76.4)	
**Do you smoke if you are so ill that you are in bed most of the day?**				
Yes	21.4 (10.2–39.5)		42.3 (25.5–61.1)	0.09
No	78.6 (60.5–89.8)		57.7 (40.0–74.5)	
**Do you use e-cigarettes if you are so ill that you are in bed most of the day?**				
Yes		34.6 (19.4–53.8)	67.9 (49.3–82.1)	0.01
No		65.4 (46.2–80.6)	32.1 (17.9–50.7)	
**FTND summary score**				
FTND cigarette score: mean ± SD	1.6 ± 1.6		3.2 ± 2.2	0.002 *
FTND e-cigarette score: mean ± SD		3.5 ± 2.6	4.7 ± 2.6	0.03 **

*95%CI indicates the 95% Confidence Interval; p* indicates the result of the U Mann-Whitney test; * indicates the result of the U Mann-Whitney test for smokers versus exclusive e-cigarette users; ** indicates the result of the U Mann-Whitney test for cigarettes versus e-cigarettes among dual users.

## Supplementary Materials

The following are available online at https://www.mdpi.com/1660-4601/16/13/2279/s1. File S1: Study questionnaire.
