# Supplementary material for: E-Cigarettes are More Addictive than Traditional Cigarettes—A Study in Highly Educated Young People
Source: Int J Environ Res Public Health. 2019 Jun 27;16(13):2279. doi: 10.3390/ijerph16132279 (PMC6651627; doi:10.3390/ijerph16132279)
Supplement: Supplementary file 1 [file ijerph-16-02279-s001.pdf]

Appendix S1 Study questionnaire.

1. Are there any addictions in your family:

[ ] Yes [ ] No

If "YES", please specify which addictions were in your family:

Chemical addictions:

a) [ ] alcohol Please indicate the relationship of the addicted person:

[ ] Parents:	[ ] mother	[ ] father
[ ] Siblings:	[ ] sister	[ ] brother
[ ] Grandparents:	[ ] grandmother	[ ] grandfather
[ ] aunt/uncle:	[ ] uncle	[ ] aunt

(I-st line parent's brothers or sisters)

b) [ ] drugs (specify?)...

Please indicate the relationship of the addicted person:

[ ] Parents:	[ ] mother	[ ] father
[ ] Siblings:	[ ] sister	[ ] brother
[ ] Grandparents:	[ ] grandmother	[ ] grandfather
[ ] aunt/uncle:	[ ] uncle	[ ] aunt

(I-st line parent's brothers or sisters)

c) [ ] nicotine (cigarettes, e-cigarettes, pipe, cigars, nicotine chewing)

Please indicate the relationship of the addicted person:

[ ] Parents:	[ ] mother	[ ] father
[ ] Siblings:	[ ] sister	[ ] brother
[ ] Grandparents:	[ ] grandmother	[ ] grandfather
[ ] aunt/uncle:	[ ] uncle	[ ] aunt

(I-st line parent's brothers or sisters)

Behavioral addiction:

a) [ ] gambling Please indicate the relationship of the addicted person:

[ ] Parents:	[ ] mother	[ ] father
[ ] Siblings:	[ ] sister	[ ] brother
[ ] Grandparents:	[ ] grandmother	[ ] grandfather
[ ] aunt/uncle:	[ ] uncle	[ ] aunt

(I-st line parent's brothers or sisters)

b) [ ] Computer games

Please indicate the relationship of the addicted person:

[ ] Parents:	[ ] mother	[ ] father
[ ] Siblings:	[ ] sister	[ ] brother
[ ] Grandparents:	[ ] grandmother	[ ] grandfather
[ ] aunt/uncle:	[ ] uncle	[ ] aunt

(I-st line parent's brothers or sisters)

2. Do you think you are addicted to any of the following:

[ ] Alcohol

[ ] Drugs (specify?)..

[ ] Nicotine (Please choose the form of nicotine delivery):

[ ] cigarette	[ ] e-cigarette	[ ] cigar
[ ] pipe	[ ] shisha	[ ] chewing nicotine

[ ] Gambling

[ ] Computer Games

3. Whether someone smoked a cigarette or an e-cigarette in your family home?

a) cigarette Yes [ ] No [ ]

"Yes", please indicate which household smoked ....

b) e-cigarette Yes [ ] No [ ]

"Yes", please indicate which household smoked ....

4. Whether in the current place of your residence someone smoked/smokes?

a) cigarette Yes [ ] No [ ]

"Yes", please indicate which household smoke ....

b) e-cigarette Yes [ ] No [ ]

"Yes", please indicate which household smoke ....

5. Is someone currently smoking a cigarette or e-cigarette in your closest surrounding?

	Cigarette	E-cigarette
a) parents	[ ]	[ ]
b) siblings	[ ]	[ ]
c) grandparents	[ ]	[ ]
d) partner	[ ]	[ ]
e) roommate	[ ]	[ ]
f) one of three closest friends	[ ]	[ ]

Questions about cigarette smoking

6. How long do you smoke ?

.... months

7. How often do you smoke ?

[ ] Everyday
[ ] 2-3 times a week
[ ] Once a week
[ ] Less than once a week

8. How many cigarettes per day do you smoke

.... cigarettes per day

9. What are the average intervals between cigarettes? (Please circle the correct one)

Minutes

1	2	3	4	5	6	7	8	9	10

20	30	40	50	60

Hours

1	2	3	4	5	6	7	8	9	10

10. . When did you stop smoking, did any of these symptoms occur?

Duration of symptom [months]

[ ] headache	1	2	3	4	5	6	7	8	9	10
[ ] irritability	1	2	3	4	5	6	7	8	9	10
[ ] anxiety in the hands	1	2	3	4	5	6	7	8	9	10
[ ] difficulty in concentratio	1	2	3	4	5	6	7	8	9	10
[ ] increased appetite	1	2	3	4	5	6	7	8	9	10
[ ] insomnia	1	2	3	4	5	6	7	8	9	10
[ ] anxiety	1	2	3	4	5	6	7	8	9	10
[ ] frequent cough	1	2	3	4	5	6	7	8	9	10

Questions about e-cigarette use

11. How long do you use e-cigarette?

.... months

12. How often do you use e-cigarette?

[ ] Everyday

[ ] 2-3 times a week

[ ] Once a week

[ ] Less than once a week

13. How many times a day do you use an e-cigarette (number of e-smoking session: one e-smoking session consist of 15 puffs or approximately 10 minutes of use) ?

.... number of e-smoking sessions

14. What are the average intervals between e-smoking sessions? (Please circle the correct one)

Minutes

1	2	3	4	5	6	7	8	9	10

20	30	40	50	60

Hours

1	2	3	4	5	6	7	8	9	10

15. When did you stop e-smoking, did any of these symptoms occur?

	Duration of symptom [months]									
[ ] headache	1	2	3	4	5	6	7	8	9	10
[ ] irritability	1	2	3	4	5	6	7	8	9	10
[ ] anxiety in the hands	1	2	3	4	5	6	7	8	9	10
[ ] difficulty in concentratio	1	2	3	4	5	6	7	8	9	10
[ ] increased appetite	1	2	3	4	5	6	7	8	9	10
[ ] insomnia	1	2	3	4	5	6	7	8	9	10
[ ] anxiety	1	2	3	4	5	6	7	8	9	10
[ ] frequent cough	1	2	3	4	5	6	7	8	9	10

16. Which type of cigarette provides greater satisfaction in selected aspects that accompany smoking?

	cigarette	no difference	e-cigarette
Feeling of relax	[ ]	[ ]	[ ]
Breathing in	[ ]	[ ]	[ ]
Breathing out	[ ]	[ ]	[ ]
Smoking satisfaction	[ ]	[ ]	[ ]
Taste	[ ]	[ ]	[ ]
Smell	[ ]	[ ]	[ ]
With a cup of coffee	[ ]	[ ]	[ ]
After meal	[ ]	[ ]	[ ]
After sex	[ ]	[ ]	[ ]
In a group of friends	[ ]	[ ]	[ ]

17. Have you noticed any changes in the last symptoms since you have used the e-cigarette?

	improvement	no difference	deterioration
Tolerance of effort	[ ]	[ ]	[ ]
Cough	[ ]	[ ]	[ ]
Tightness of the chest	[ ]	[ ]	[ ]
Wheezing, squeaks in the chest	[ ]	[ ]	[ ]
Beathlessness	[ ]	[ ]	[ ]
Dizziness	[ ]	[ ]	[ ]
Weakness	[ ]	[ ]	[ ]
Headache	[ ]	[ ]	[ ]
Scratching in the throat	[ ]	[ ]	[ ]
Smell	[ ]	[ ]	[ ]
Taste	[ ]	[ ]	[ ]
Dry mucous membranes	[ ]	[ ]	[ ]

18. If you are currently smoking traditional cigarettes, please answer the following questions:

How soon after waking up do you reach for a cigarette	[ ] within 5 min<br>[ ] 6-30 min<br>[ ] 31-60 min<br>[ ] after 60 min
How many cigarettes per day do you smoke?	[ ] 10 or less<br>[ ] 11-20<br>[ ] 21-30<br>[ ] 31 or more
Do you find it difficult to refrain from smoking in places where it is forbidden?	[ ] Yes<br>[ ] No
Which cigarette would you hate most to give up?	[ ] the first one in the morning<br>[ ] any other
Do you smoke more frequently during the first hours after waking than during the rest of the day??	[ ] Yes<br>[ ] No
Do you smoke if you are so ill that you are in bed most of the day?	[ ] Yes [ ] No

19. <u>If you currently use an e-cigarette, please answer the following questions:</u>

How soon after waking up do you reach for an e-cigarette	[ ] within 5 min<br>[ ] 6-30 min<br>[ ] 31-60 min<br>[ ] after 60 min
How many times a day do you use e-cigarette? (e-smoking sessions; one e-smoking session consist of 15 puffs or approximately 10 minutes of use)	[ ] 10 or less<br>[ ] 11-20<br>[ ] 21-30<br>[ ] 31 or more
Do you find it difficult to refrain from e-smoking in places where it is forbidden?	[ ] Yes<br>[ ] No
Which e-smoking session would you hate most to give up?	[ ] the first one in the morning<br>[ ] any other
Do you use e-cigarette more frequently during the first hours after waking than during the rest of the day?	[ ] Yes<br>[ ] No
Do you use e-cigarette if you are so ill that you are in bed most of the day?	[ ] Yes [ ] No

Questions about e-smoking habits

20. Questions about e-liquid

a) E-liquid Manufacturer (please write the most common used brand or if you seld-made e-liquid, please note that) ....

b) Most frequently used taste of e-liquid: ....

c) Nicotine concentration in e-liquid (%):...

d) E-liquid consumption (ml) daily: .... monthly: ....

21. Have you ever modified the technical parameters of the e-cigarette? Yes No

22.Have you ever used a self made e-liquid? Yes No

23.How many different models of e-cigarette you used:

24.Where did you find out about the e-cigarette:

25. Source of knowledge about e-cigarettes (mark all appropriate):

[ ] Internet forums
[ ] E-cigarette users discussion groups on Facebook
[ ] Websites of e-cigarette shops
[ ] Blogs about e-cigarettes
[ ] Friends who use e-cigarette
[ ] Newspapers for e-smokers
[ ] Other (please specify).....................
